# Assessing causality in epidemiology: revisiting Bradford Hill to incorporate developments in causal thinking

**DOI:** 10.1007/s10654-020-00703-7

**Published:** 2020-12-16

**Authors:** Michal Shimonovich, Anna Pearce, Hilary Thomson, Katherine Keyes, Srinivasa Vittal Katikireddi

**Affiliations:** 1 MRC/CSO Social and Public Health Sciences Unit, University of Glasgow, Glasgow, UK; 2 Mailman School of Public Health, Columbia University, New York, NY, USA

**Keywords:** Causal inference, Bradford Hill, Directed acyclic graphs, Sufficient component cause models, GRADE

## Abstract

The nine Bradford Hill (BH) viewpoints (sometimes referred to as criteria) are commonly used to assess causality within epidemiology. However, causal thinking has since developed, with three of the most prominent approaches implicitly or explicitly building on the potential outcomes framework: directed acyclic graphs (DAGs), sufficient-component cause models (SCC models, also referred to as ‘causal pies’) and the grading of recommendations, assessment, development and evaluation (GRADE) methodology. This paper explores how these approaches relate to BH’s viewpoints and considers implications for improving causal assessment. We mapped the three approaches above against each BH viewpoint. We found overlap across the approaches and BH viewpoints, underscoring BH viewpoints’ enduring importance. Mapping the approaches helped elucidate the theoretical underpinning of each viewpoint and articulate the conditions when the viewpoint would be relevant. Our comparisons identified commonality on four viewpoints: strength of association (including analysis of plausible confounding); temporality; plausibility (encoded by DAGs or SCC models to articulate mediation and interaction, respectively); and experiments (including implications of study design on exchangeability). Consistency may be more usefully operationalised by considering an effect size’s transportability to a different population or unexplained inconsistency in effect sizes (statistical heterogeneity). Because specificity rarely occurs, falsification exposures or outcomes (i.e., negative controls) may be more useful. The presence of a dose-response relationship may be less than widely perceived as it can easily arise from confounding. We found limited utility for coherence and analogy. This study highlights a need for greater clarity on BH viewpoints to improve causal assessment.

## Introduction

Causal assessment is fundamental to epidemiology as it may inform policy and practice to improve population health. A leading figure in epidemiology, Sir Austin Bradford Hill, suggested the goal of causal assessment is to understand if there is “any other way of explaining the set of facts before us … any other answer equally, or more, likely than cause and effect” [1]. Causal assessment may be applied to a body of evidence or a single study to interrogate the “set of facts” underlying a relationship. Bradford Hill notably laid out a set of such facts. Although commonly described as Bradford Hill criteria, he described them as ‘viewpoints’ and emphasised they should not be used as a checklist, but as considerations for assessing causality. As a result, we refer to them as ‘BH viewpoints’ [2].

Since Bradford Hill first introduced his viewpoints, causal thinking in epidemiology has increasingly incorporated the potential outcomes framework [3–8]. Informally, the potential outcomes framework posits that a true causal effect is the difference between the *observed* outcome when the individual was exposed and the *unobserved* potential outcome had the individual not been exposed, all other things being equal [6]. Because the unobserved potential outcome of an individual cannot be known, investigators often compare the outcomes of exposed and unexposed groups [6]. Application of the potential outcomes framework asks investigators to consider exchangeability between these groups i.e., if the unexposed group would have the same risk of the outcome as the exposed group had they also been exposed [6]. In practice, this means considering if groups are comparable. Investigators may be more confident that the observed effect equals the true causal effect if the groups are exchangeable [9].

We focus on three approaches that implicitly or explicitly incorporate the potential outcomes framework but operationalise it differently [4, 10–12]. Firstly, directed acyclic graphs (DAGs) help articulate assumptions about the inter-relationships between variables of interest and therefore threats to valid causal inference. Sufficient-component cause (SCC) models highlight the multi-factorial nature of causality, drawing attention to how different exposures interact to produce the outcome. Finally, the Grading of Recommendations, Assessment, Development and Evaluation (GRADE) methodology provides a systematic approach to assessing the certainty of a causal relationship based on a body of evidence (i.e., the existing studies available used to assess whether a causal relationship between an exposure and outcome exists). Epidemiologists have proposed that causal assessment may be improved by combining approaches such as these [7, 13–15].

To draw on the strengths of each of these potential outcomes framework approaches, we compared the extent to which they overlap or complement each other. There is limited literature comparing the potential outcomes framework in SCC models and DAGs [4, 5, 11] and one study comparing BH viewpoints to GRADE [10]. While BH viewpoints have been revisited to critically reflect on the theory and application of each viewpoint [2, 16–20], we have not identified any attempts to compare it to DAGs and SCC models, with the former particularly important given the growing influence of DAGs in epidemiology [21].

Our main aims are to examine: 1) if and how each BH viewpoint is considered by each of the three potential outcomes framework approaches (referred to simply as ‘approaches’ hereafter); and 2) the extent they elucidate the underpinning theory of BH viewpoints. BH viewpoints serve as the foundation for this comparison because of its influential status within epidemiology [19, 20, 22]. Additionally, there is agreement in the literature that the BH viewpoints account for the most relevant considerations in causal assessment [17]. To facilitate comparisons, we drew DAGs and SCC models for each BH viewpoint and mapped each BH viewpoint against each GRADE domain. We use the example of alcohol consumption and active-tuberculosis where relevant to illustrate the elements of each approach. *Mycobacterium tuberculosis* (MTB) is the bacterium responsible for tuberculosis (TB). MTB causes latent-TB, which can turn into active-TB in individuals with low immunity [23]. Alcohol consumption is hypothesised to cause a weaker immune system, resulting in active-TB [24]. The example is purposefully simplified and may not reflect real-world scenarios.

In the next section, we summarise the BH viewpoints and key characteristics of the three approaches they are being compared against. Our aim is to introduce the commonalities and distinctions within these approaches as approaches to causal inference, rather than to provide a detailed explanation or critical assessment of each approach. Following this, we compare each of the nine BH viewpoints against the three approaches and critically reflect on the theoretical implications for assessing causal relationships. We finish by summarising our key findings, make tentative suggestions about how causal assessment could be conducted in the future and note some areas for future research.

## Causal assessment approaches

### Bradford Hill viewpoints

Bradford Hill’s explanation of the nine viewpoints is summarised in Table 1. These were not intended to be “hard and fast rules of evidence that must be obeyed before we accept cause and effect,” but characteristics to keep in mind while considering if an observed association is due to something other than causality [1]. In current practice, BH viewpoints are applied together or separately to a body of evidence or a single empirical study.

### Directed acyclic graphs

DAGs are diagrams that illustrate the putative causal relationship between an exposure and outcome [6]. DAGs include the variables that might bias the relationship in question and their development is based on background knowledge of the topic [25]. Detailed explanations of DAGs can be found elsewhere [5, 6, 25–27]. DAGs are commonly applied to a single study, but it has been proposed that they can be applied to a body of evidence [62].

**Fig. 1:**
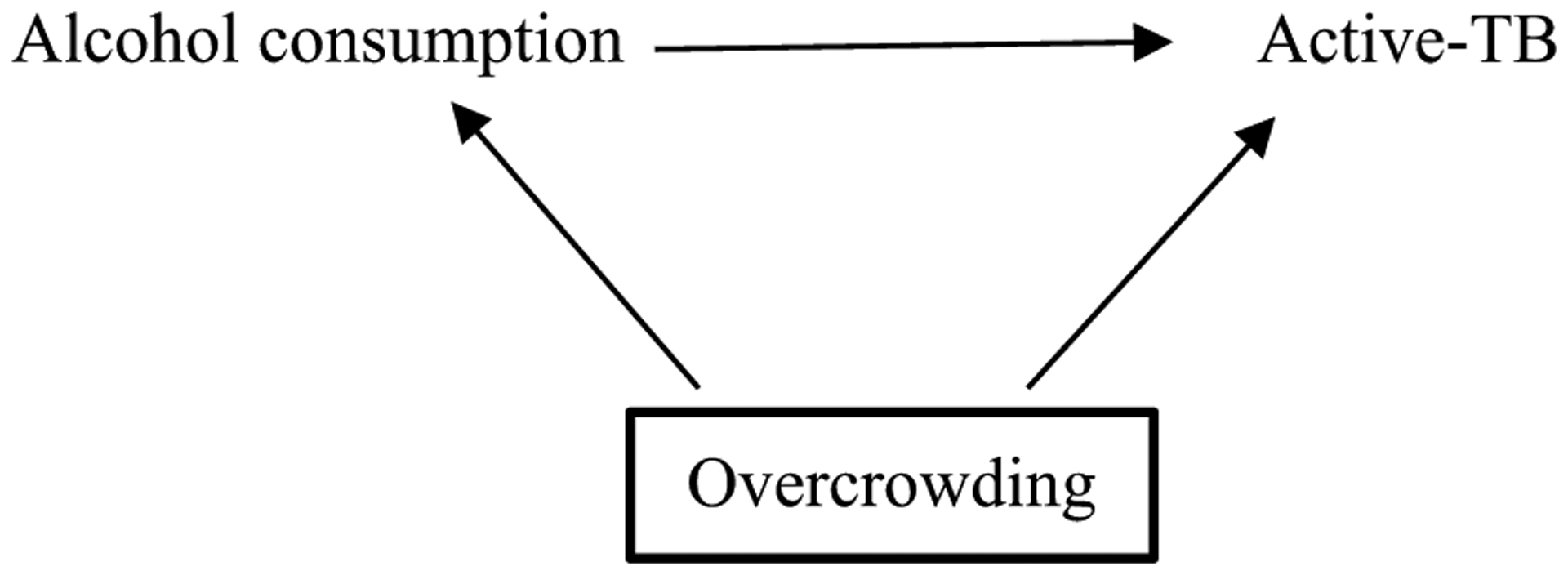
Directed acyclic graph representing relationship between alcohol consumption and active-TB. The confounding variable, overcrowding, effects both the exposure and outcome and should be conditioned on, as indicated by the bold square around overcrowding

The simplified DAG below (Fig. 1) shows the pathway between the exposure and outcome, alcohol consumption and active-TB, respectively. Alcohol consumption may result in active-TB, for example, by lowering an individual’s immune system (mediator not shown) [23]. Overcrowding is a confounding variable, causing both alcohol consumption and active-TB. If there was no causal effect of alcohol consumption on active-TB (i.e. no edge between those two variables in the DAG), an association would still be observed between them in the data due to the common cause overcrowding [4, 25, 28, 29]. Thus, overcrowding must be conditioned upon, indicated by a square around the variable, to obtain an unbiased estimate of alcohol consumption on active-TB. If investigators condition on the appropriate variables using a DAG that accurately represents a causal relationship, they may be more confident of exchangeability and thus estimating the true causal effect [9, 30].

### Sufficient-component cause (SCC) models

SCC models (also known as causal pies) illustrate the multi-factorial nature of causality through pie charts [31]. SCC models view each of the variables that contribute to the outcome occurring as causal components [32], with many different combinations of components potentially bringing about the outcome of interest. Taken together, the components for each ‘complete pie’ are *sufficient* to produce the outcome. *Necessary* components are those without which the outcome could not occur [33]. For example, MTB is a necessary (but insufficient) component of tuberculosis and will therefore be a component for all of the causal pies for tuberculosis (but never features as a sole component of a causal pie). The origins of SCC models can be traced to Mackie’s definition of causality. This introduced the idea of INUS causation, that is a cause can be “an *insufficient* but *necessary* part of a condition which is itself *unnecessary* but *sufficient* for the result” [34] p. 45.

Causal pies are useful for understanding causal mechanisms and interactions of causal components [33]. Table 2 illustrates four pies (*S*_*1*_, *S*_*2*_, *S*_*3*_, *S*_*4*_) for two different populations (population 1 and population 2) which represent the possible combination of selected causal components (alcohol, overcrowding and unknown factors) for the development of active TB.

### GRADE methodology

GRADE is the most widely adopted approach for assessing certainty of evidence in systematic reviews, guideline development and evidence-informed recommendations [35]. Certainty has been defined by the GRADE Working Group as the “extent of our confidence that the estimates of the effect are correct” [10, 36–38]. Certainty is based both on assessing the risk of bias of individual studies and an evaluation *across* studies [35]. GRADE typically considers evidence from randomised controlled trials (RCTs) as providing a higher level of certainty than evidence from nonrandomised studies (NRSs), although the appropriateness of this has been critiqued [39]. Certainty may be modified according to different GRADE domains (summarised in Table 3). Large associations, dose-response relationships and adjusting for plausible confounding upgrade certainty.

## Comparisons against Bradford Hill’s viewpoints

Table 4 summarises the overlapping elements between BH viewpoints and the potential outcomes framework approaches, with subsequent text providing additional detail.

### Strength of association

Bradford Hill argued that a large association suggests the observed effect is less likely to be due to bias [1, 40], but he acknowledged that weak (or small) associations may still reflect causal relationships. As noted by Greenland and Robins, large associations can still arise from confounding and a weak association does not mean there is an absence of causality[33]. In practice, investigators may rely on existing tools and guidelines, or their own interpretation, to determine what constitutes a strong association.

Although DAGs cannot represent the size of an association, they facilitate “bias analysis” (see Fig. 1) [14]. Investigators may use DAGs to highlight important variables that they were unable to condition on and consider their implications for the effect estimate, including residual confounding (from inaccurately or poorly measured variables, including confounders) [41].

SCC models draw attention to the impact of disease prevalence and the prevalence of competing causes on the strenth of association or effect estimate. For example, the RR of S_3_ is attenuated as the prevalence of a competing sufficient cause (S_4_) or the prevalence of the outcome in the reference group (S_1_) increases (see Table 2).

According to the GRADE Working Group, a strong association is indicated by a risk ratio (RR) of 2–5 or 0.2–0.5 [17, 17, 17]. Evidence from NRSs that estimate a large effect will be upgraded on the basis that confounding is less likely to entirely remove the observed association [43].

### Consistency

Bradford Hill argued that consistent estimates observed in different circumstances reduce the likelihood that the effect is due to chance or bias [1]. Comparison with the three approaches demonstrate that differences in effect size across studies which may be due to variations in causal structures, variable interactions, or biases of the relevant studies.

**Fig. 2:**
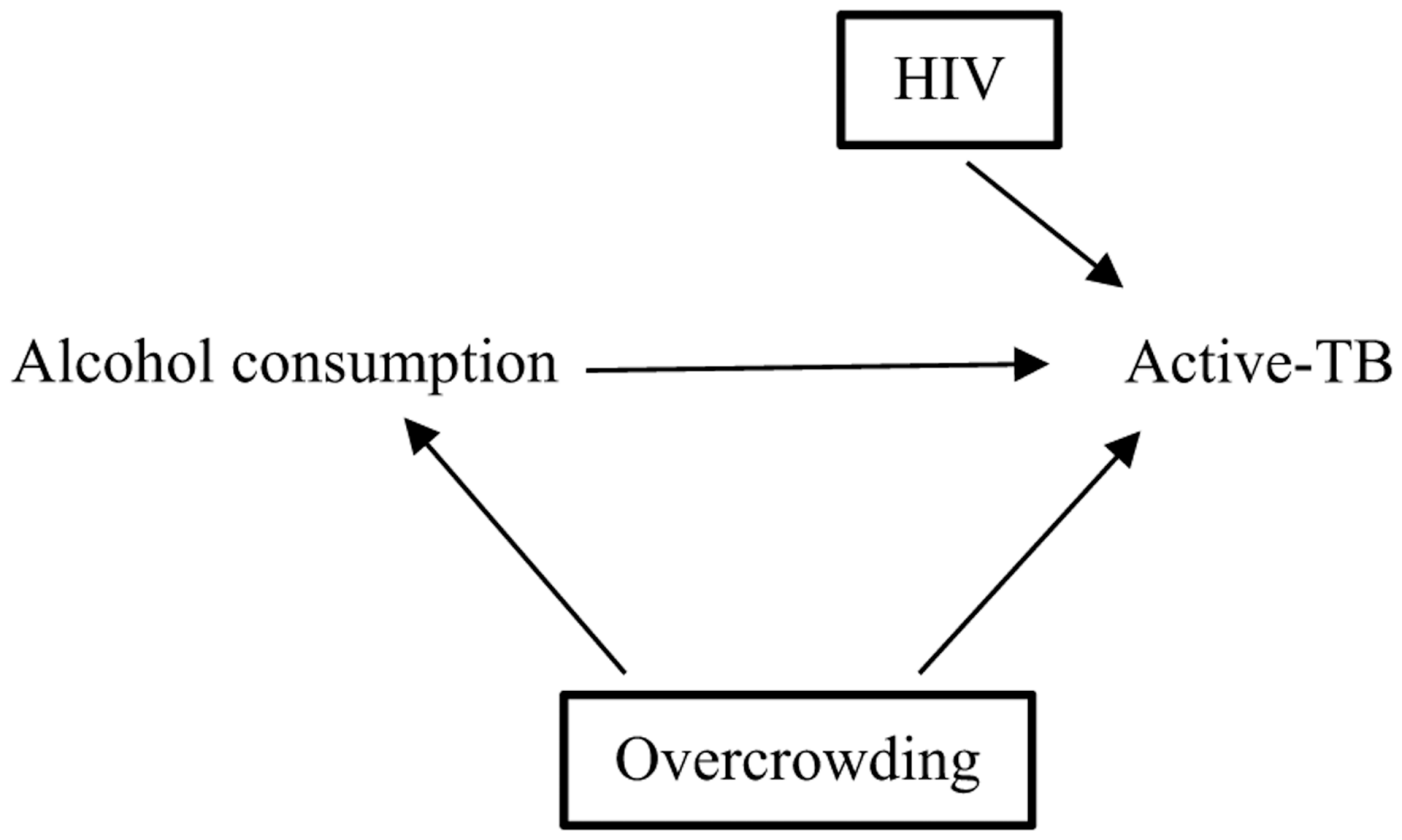
Directed acyclic graph (DAG) of target population with high baseline risk of HIV. The high baseline risk of HIV means that HIV has been conditioned upon, indicated by square around HIV. The estimated effect of alcohol consumption on active-TB in this population will be modified by the higher risk of HIV. This needs to be considered when comparing the effect estimates between this target population and the one described in Fig. 1 with low risk of HIV

Transportability refers to the extent to which a causal effect in one context can be used to infer a causal effect in different circumstances, such as different populations or study designs [44]. Investigators can use DAGs to understand how differences in causal structures may explain different observed effect sizes. For example, investigators may want to understand if the causal effect of alcohol consumption on active-TB can be extrapolated to a target population with a high baseline risk of HIV (represented in Fig. 2). In other words, to understand if the different effect size in the target population is due to HIV modifying the effect of alcohol consumption on active-TB by reducing immunity [45, 46]. To represent the target population’s exposure to a stratum of HIV (i.e., a higher risk of HIV), there is a square around HIV [44, 46]. If the likelihood of active-TB for a given level of alcohol consumption is equivalent between the populations, the estimated effect of alcohol on active-TB is transportable and any statistical heterogeneity observed is likely due to HIV risk modifying the effect of alcohol on active-TB[46].

Investigators can use SCC models to understand differences in variable interactions and if that can explain different observed effect sizes observed between populations [44, 47–49]. For example, investigators may want to understand if the RR of individuals in population 1 in Table 2 can be transported to population 2. According to Table 2, the RR of active-TB when individuals are exposed *only* to overcrowding (S_3_) is lower in population 2 than population 1. i.e., the effect of overcrowding on active-TB differs between populations when alcohol is not consumed. It may be that the unknown factors of S_3_ differ between populations. However, because the RRs are the same for other causal pies, investigators may assume that the reason for different prevalence and RRs for S_3_ is that unknown factors and overcrowding are interacting differently between the populations, in which case the effect sizes cannot be transported from population 1 to population 2.

In GRADE, unexplained inconsistency (typically, statistical heterogeneity) suggests lower confidence about the likely effect of the exposure under different circumstances. GRADE considers unexplained inconsistency rather than consistent effect estimates, as Bradford Hill suggested, to highlight that consistent estimates in different circumstances may be subject to the same bias and do not necessarily increase confidence in causality [50].

### Specificity

According to Bradford Hill, a relationship is specific if the exposure is associated with the outcome in question and no others, *and* if the outcome is associated with the exposure in question and no others. He emphasised that a non-specific relationship does *not* undermine causality. Specificity originated in Robert Koch’s postulates to evaluate causality in infectious diseases, but is rare in epidemiology and usually arises when the outcome is defined based on the exposure status (e.g., tuberculosis being defined by the presence of the tubercle bacillus) [17, 51, 52]. Comparisons highlighted how multiple causation (where one exposure may affect many outcomes and one outcome may be effected by many exposures) limits the utility of directly applying specificity in epidemiological practice, but extending the concept to the related idea of ‘falsification’ may improve its usefulness.

The DAG in Fig. 1 illustrates a non-specific relationship as active-TB is caused by at least two exposures: alcohol-consumption and overcrowding [53]. The relationship is also non-specific because alcohol consumption may cause many other outcomes such as cancer, cardiovascular disease and injuries [54]. This is not shown in the DAG in Fig. 1 because DAGs typically include the main variables related to the relationship of interest (i.e., an exposure, outcome and any potential confounders) [55]. This is also the reason why DAGs are not used to demonstrate specific relationships; a variable may be left out of a DAG because it is not of interest, not because the relationship illustrated in the DAG is specific.

One important reason for specificity is multiple causation suggests a higher likelihood that the observed association is due to confounding. Rather than seeking evidence of specificity, DAGs can be used to help identify and assess falsification (or negative control) outcomes and exposures. A falsification outcome is expected to be both independent of the outcome and associated with the exposure only through the confounding variable [56]. If investigators accurately condition on the confounding variable, they would not observe an effect of the exposure on the falsification outcome.

**Fig. 3:**
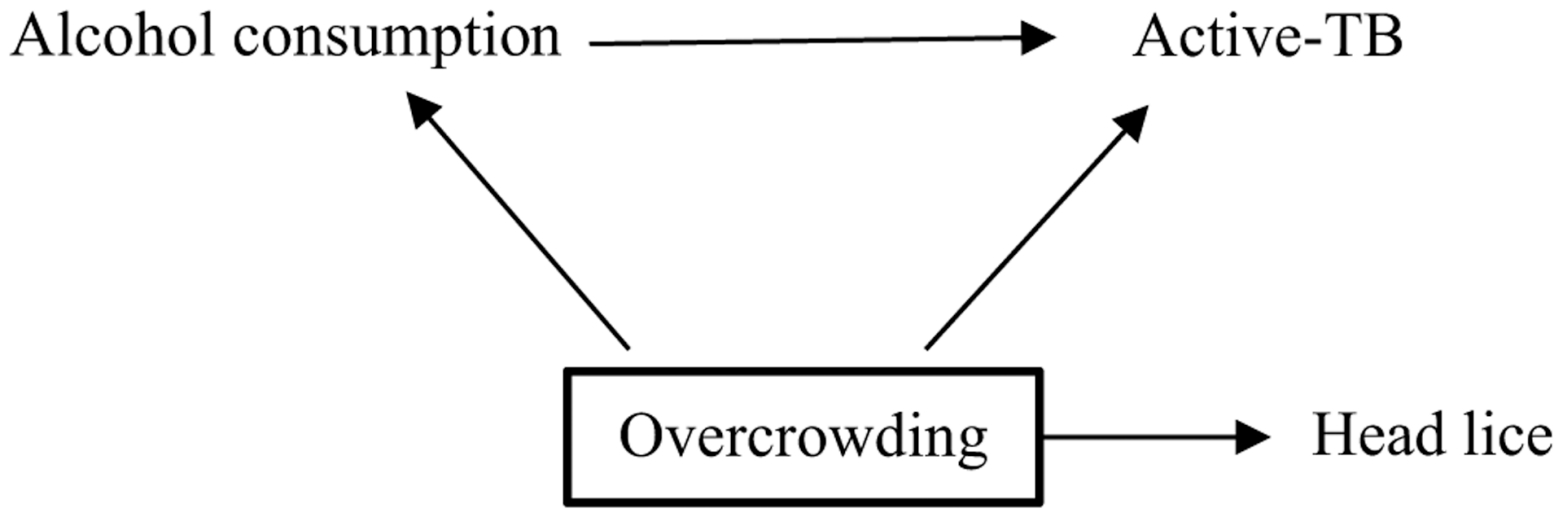
The directed acyclic graphs (DAG) shows the relationship between the exposure (alcohol consumption), the outcome (active-TB), the confounding variable (overcrowding) and the falsification outcome (head lice). The bold square around overcrowding indicates that it has been conditioned on. If there is no effect of alcohol consumption on head lice, there is a greater likelihood that overcrowding has been accurately conditioned upon

A hypothetical falsification outcome is head lice (Fig. 3). Alcohol consumption does not have a causal effect on head lice. If investigators observe an effect of alcohol consumption on head lice despite conditioning upon overcrowding, this is likely due to residual confounding due to overcrowding being inaccurately measured. Therefore, it is possible that the relationship between alcohol and active-TB is also subject to residual confounding of overcrowding and investigators should adjust their conclusions accordingly. An absence of association between alcohol consumption and head lice does not suggest specificity, but investigators may be more confident that in this study, the association between alcohol consumption and active-TB is not confounded by overcrowding.

Finding falsification variables can be challenging. Take the example of identifying a falsification exposure (which is independent of the exposure and associated with the outcome only through the confounding variable). Many possible exposures associated with the confounder (overcrowding), such as smoking, air pollution, experiences of homelessness and malnutrition are also associated with the outcome (active-TB) and therefore would fail as a falsification exposure [57, 58]. Put another way, the lack of specificity in most causal relationships in epidemiology limits our ability to carry out falsification tests. However, where they do exist they can offer a powerful tool for assessing bias.

Causal pies illustrate the multi-factorial nature of causal relationship that limits the likelihood of specificity because a range of causal pies (and causal components) may produce the same outcome (see Table 2). One causal pie may also be used to represent a possible sufficient-cause for various exposures[59]. The causal pie would represent a specific relationship only if a component is both necessary and sufficient to produce the outcome and the outcome could only be produced by this necessary and sufficient cause [31, 33]. These limitations are among the reasons why some, including the originators of GRADE methodology, argue that specificity should be excluded from causal assessment [7, 10, 31, 60].

### Temporality

Temporality is considered fundamental to causality; an exposure must precede an outcome. Bradford Hill alluded to how reverse causality skews temporality: “does a particular occupation or occupational environment promote infection by the tubercle bacillus … or, indeed, have they already contracted it?” [1]. Two of the three approaches explicitly incorporate temporality, with the order of cause and effect being fundamental to DAGs.

**Fig. 4:**
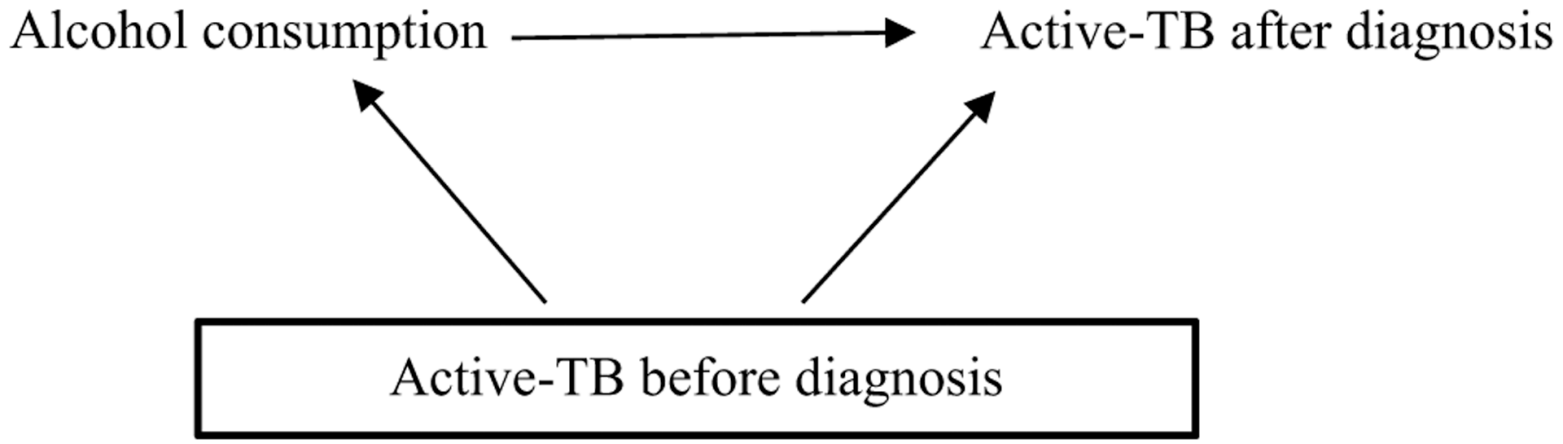
Temporality using directed acyclic graphs (DAGs). Investigators may be more confident that the effect of alcohol consumption on active-TB is not due to reverse causality if (1) they condition upon active-TB before diagnosis and continue to observe an effect of alcohol consumption on active-TB after diagnosis or (2) if they do not observe an effect of active-TB before diagnosis on alcohol consumption

DAGs can highlight reverse causality [20, 61]. For example, in a cross-sectional study, the observed effect of alcohol consumption is based on measurements after individuals were diagnosed with active-TB. However, active-TB may have actually occurred prior to *diagnosis* of active-TB and been a cause of alcohol consumption, via social marginalisation [62]. Given a longitudinal study that has information on previous diagnoses, investigators could test for reverse causation by considering if active-TB was present *before* the diagnosis that was observed after alcohol consumption (see Fig. 4). If investigators conditioned upon active-TB before diagnosis and continued to observe an effect of consuming alcohol on active-TB *after* diagnosis, or if they found no effect of active-TB before diagnosis on alcohol consumption, then the estimated effect of alcohol consumption on active-TB after diagnosis is less likely due to reverse causation.

Time may be one component of a causal pie but temporality is not considered in the synergy, antagonism and interaction of the components [2]. Temporality is not directly considered by GRADE. RCTs, which guarantee that the exposure precedes the outcome through study design, are upgraded. However, the favouring of RCTs is not only about temporality but also about the achievement of exchangeability through randomisation. Additionally temporality is not explicitly considered for NRSs (which include longitudinal studies and so may also be able to ensure that the exposure precedes the outcome).([10].

### Dose-response

A dose-response gradient exists when incremental increases (or decreases) of the exposure produce incremental increases (or decreases) of the outcome. Dose-response is fundamental to causal assessment in pharmacology and toxicology [63]. Bradford Hill argued that a dose-response gradient provides a “simpler explanation” of the causal relationship than if it were not observed (see Table 1) [1]. However, there are many reasons investigators may not observe a dose-response gradient including exposure threshold effects, as in the case of allergens [17]. Furthermore, a dose-response relationship may be induced by a confounding variable [64, 65]. For example, an incremental increase in alcohol consumption that corresponds to an incremental increase in active-TB may be due to incremental increases in overcrowding (see Fig. 1) [66]. While DAGs non-parametric (and so cannot show the structure of the relationship between any two variables), they can be used to consider the plausibility of one or more confounding variables undermining a dose-response relationship.

Unknown components limit the utility of SCC models to assess dose-response gradients. Evidence from NRSs is upgraded in GRADE if a dose-response relationship has been observed on the basis that confounding is less likely [35]. However, as noted above, a dose-response relationship may easily arise from confounding.

### Plausibility

Investigators develop assumptions about a causal relationship based on background knowledge. Thus, the plausibility of the causal relationship is both dependent on and limited by knowledge available at the time [1]. It may be further limited by assumptions based on investigators’ beliefs rather than empirical evidence [67].

The process of developing DAGs and SCC models forces investigators to explicitly articulate assumptions about the causal relationships relevant to the research question of interest, making it transparent to other investigators [44, 68] [69]. DAGs may include mediators, which lie on the causal path between the exposure and outcome; a weakened immunity is the mediator by which alcohol consumption causes active-TB. Mediation analysis considers the direct and indirect effect of mediators [70]. Interrogating background knowledge to develop a DAG encourages a more systematic exploration of the plausibility of the causal chain.

For SCC models, investigators make explicit the nature of variable interaction [71]. GRADE upgrades for appropriate adjustment for all plausible confounding variables, but does not consider the broader variables relevant to the plausibility of a causal relationship across a body of evidence [35].

### Coherence

Coherence is an assessment of how the putative relationship fits into existing theory and empirical evidence [1, 60]. Our comparisons suggest that coherence is not considered by the other approaches and may have limited utility, partly because it is poorly delineated from plausibility [72]. Investigators evaluating the coherence of a DAG or SCC model may consider how the assumptions illustrated by either approach fit existing theory, however, neither consider or illustrate coherence. Schünemann and colleagues argue that GRADE considers coherence by assessing indirectness [10]. However, in considering indirectness, investigators determine how applicable the population and interventions of identified studies are to the putative causal relationship under study. Coherence, on the other hand, asks investigators to consider how applicable the putative causal relationship is to broader evidence, including studies that do not investigate that specific relationship.

### Experiment

Bradford Hill argued that “strong support for the causation hypothesis might be revealed” from “experimental, or semi-experimental data” [1]. He alluded to natural experiment studies, where the exposure is determined by nature or other factors outside of the control of investigators and where exchangeability between comparison groups is more likely [29].

**Fig. 5:**
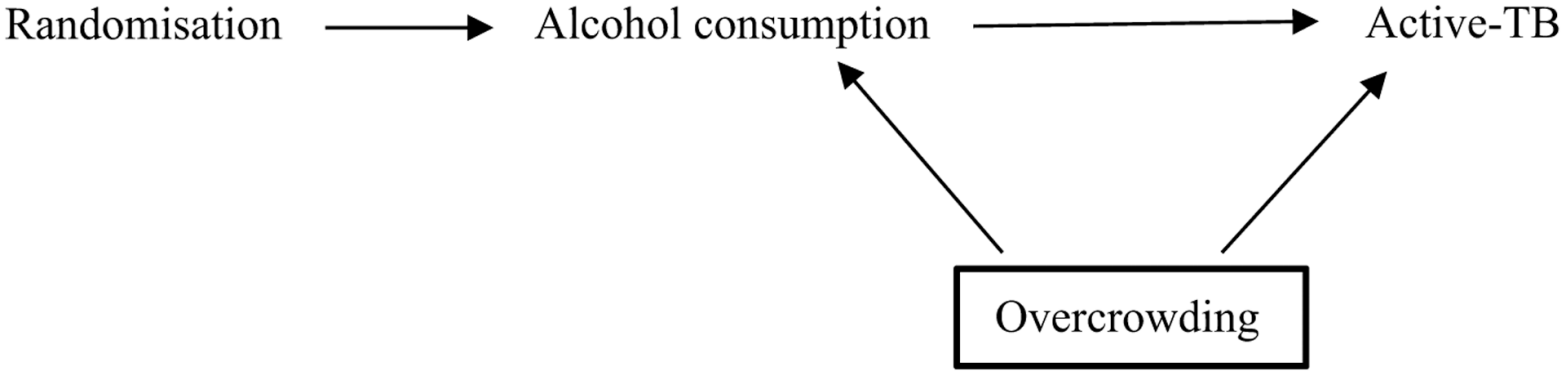
Directed acyclic graph (DAG) with randomisation as the instrumental variable. According to this DAG, randomisation causes alcohol consumption. If this were true, there is a greater likelihood that the effect estimated would be similar or equivalent to the true causal effect

Investigators have used DAGs to elucidate why randomisation results in exchangeability. Randomisation is an example of an instrumental variable; it causes (and is not caused by) the exposure and only impacts the outcome through the exposure [73]. If consuming alcohol was completely random and randomisation was independent of active-TB (see Fig. 5), the risk of overcrowding would be the same for individuals allocated to consume alcohol and those allocated to not [74]. Thus, the effect estimated would be based on exchangeable groups, but bounded by the proportion of individuals exposed due to randomisation, potentially limiting the transportability of the effect estimate [44, 75].

Due to limitations on randomisation, epidemiologists rely largely on observational data. Investigators can use DAGs to interrogate the plausibility of “naturally occurring” instrumental variables, and how likely it is that individuals were truly randomly exposed [29, 73]. Clarity about study design, particularly procedures for assigning exposure, has been assisted by DAGs through the development of the ‘target trial’ (or ‘emulated trial’) where observational data analysis emulates randomised trial data analysis [76]. While it has several advantages, this does not seem to be directly comparable with the original BH viewpoint.

The causal pies that result in a given disease include both known and unknown components, as shown in Table 2. As investigators are unable to measure unknown variables for each causal pie, they cannot be certain that the groups exposed to each causal pie are exchangeable because they may differ in other characteristics that affect the outcome [4, 11]. GRADE privileges effect estimates from randomised (experimental) studies which are more likely to be “causally attributable to the intervention” by initially grading RCTs higher than NRSs [43]. At present, no distinction is made between natural experiment studies and other NRSs on the basis of study design.

### Analogy

Bradford Hill argued that the likelihood of a causal relationship may be strengthened if a comparable association is observed between the same outcome and an analogous exposure or the same exposure and an analogous outcome. DAGs and SCC models do not account for analogous relationships in their assessment, but analogous relationships may be part of developing the assumptions and theories encoded in the diagrams. In GRADE, downgrading would be prevented if there was certainty in a causal relationship between the same exposure and similar outcomes in the same body of evidence [10]. While this has been conflated with analogy, this is more to do with the directness of the evidence to the research question rather than the transportability of the assumptions of an analogous, confirmed causal relationship to the one under study [77].

## Discussion and conclusions

Epidemiologists evaluate evidence to understand how likely it is the observed effect is equal to the causal effect. We mapped DAGs, SCC models and GRADE against each BH viewpoint by comparing each tool to identify the overlap between different perspectives on causal assessment. The summary of these comparisons and the potential implications for causal assessment can be found in Table 5.

The comparisons highlight the overlap between BH viewpoints and other approaches. This underscores the ongoing influence of BH viewpoints in causal assessment alongside developments in causal thinking. It also highlights the importance of other approaches in understanding BH viewpoints. DAGs help explain the theoretical underpinning of strength of association, consistency, temporality, specificity, dose-response, plausibility, and experiment. GRADE provides guidance on how causal assessment can be applied in practice, particularly for considering strength of association, consistency, temporality, dose-response and experiment. While the inclusion of SCC models can be debated as they can be considered a framework to describe causal reality and are least used of the approaches we studied, their inclusion has been useful for understanding strength of association and plausibility in our analysis. Despite their seemingly limited utility for understanding BH viewpoints, SCC models, along with GRADE, also help explain why specificity may have limited usefulness in causal inference.

Our analysis is the first to compare insights from advancements in causal assessment with BH viewpoints [7]. This is an area that requires further research and we hope our study will encourage debate and discussion on overlapping approaches to causal inference. Further research and discussion is necessary to develop a new and comprehensive set of causal criteria that incorporates both traditional and recently developed approaches in causal inference. Such work would likely benefit from applying these different approaches to specific research questions, with a view to identifying their relative capacity to facilitate causal assessment. However, we did not critique the individual approaches as this has been done in previous works [4, 5, 10, 11]. We did not investigate all potential approaches to assessing causality (e.g. International Agency for Research on Cancer and criteria for teratogenicity) due to limited time and resources. Instead, we focused on GRADE, DAGs and SCC models which are perhaps the best-known causal assessment approaches outside of BH viewpoints.

This study underscores the need for greater clarity on causal assessment in epidemiology. This is an initial attempt to demonstrate how recent approaches can be used to elucidate BH viewpoints, which remain fundamental to causal assessment and to tentatively suggest how their application could be improved. Our findings are preliminary and we welcome debate about our comparisons and the suggested implications for causal assessment.

## Figures and Tables

**Table 1:** Bradford Hill viewpoints and explanatory quotations

Viewpoint	Explanatory quotations from Bradford Hill [1]
Strength of association	“But to explain the pronounced excess in cancer of the lung *[among cigarette smokers]* in any other environmental terms requires some feature of life so intimately linked with cigarette smoking and with the amount of smoking that such a feature should be easily detectable.” p. 296
Consistency	“We have, therefore, the somewhat paradoxical position that the different results of a different inquiry certainly cannot be held to refute the original evidence; yet the same results from precisely the same form of inquiry will not invariably greatly strengthen the original evidence. I would myself put a good deal of weight upon similar results reached in quite different ways, e.g. prospectively and retrospectively.” p. 296–297
Specificity	“If, as here, the association *[between working as a nickel refiner and cancer]* is limited to specific workers and to particular sites and types of disease and there is no association between the work and other modes of dying, then clearly that is a strong argument in favour of causation. We must not, however, over-emphasize the importance of the characteristic *[specificity].”* p. 297
Temporality	“Which is the cart and which the horse? This is a question which might be particularly relevant with diseases of slow development.” p. 297
Dose-response	“For instance, the fact that the death rate from cancer of the lung rises linearly with the number of cigarettes smoked daily, adds a very great deal to the simpler evidence that cigarette smokers have a higher death rate than non-smokers.” p. 298
Plausibility	“But this is a feature I am convinced we cannot demand. What is biologically plausible depends upon the biological knowledge of the day.” p. 298
Coherence	“On the other hand, the cause-and-effect interpretation of our data should not seriously conflict with the generally known facts of the natural history and biology of the disease.” p. 298
Experiment	“Occasionally it is possible to appeal to experimental, or semi-experimental, evidence. For example, because of an observed association some preventive action is taken. Does it in fact prevent? The dust in the workshop is reduced, lubricating oils are changed, persons stop smoking cigarettes. Is the frequency of the associated events affected? Here the strongest support for the causation hypothesis may be revealed.” p. 298–299
Analogy	“In some circumstances it would be fair to judge by analogy. With the effects of thalidomide and rubella before us we would surely be ready to accept slighter but similar evidence with another drug or another viral disease in pregnancy.” p. 299

**Table 2:** Sufficient component cause models and corresponding prevalence rates and risk ratios (RRs) for each sufficient-cause between two populations

Column 1: Causal pies	2: Alcohol consumption	3: Overcrowding	Population 1				Population 2			
			Prevalence of outcome for each sufficient-cause				Prevalence of outcome for each sufficient-cause			
			4: Active-TB	5: Not-active-TB	6: Risk of active-TB	7: Risk ratio (RR)	8: Active-TB	9: Not-active-TB	10: Risk of active-TB	11: Risk ratio (RR)
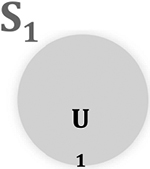	0	0	20	80	0.2	Reference group	20	80	0.2	Reference group
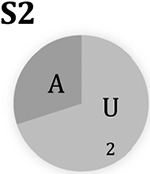	1	0	60	40	0.6	3.0	60	40	0.6	3.0
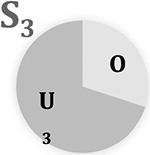	0	1	70	30	0.7	3.5	40	60	0.4	2.0
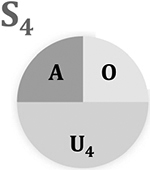	1	1	90	10	0.9	4.5	90	10	0.9	4.5

The prevalence of each causal pie differs in each population, and as a result the RR differs in each population

Unknown factors may differ in each combination of components, as indicated by the different subscripts of *U* corresponding to each SCC model. In a hypothetical dataset of 400 individuals, A and O are measured and U is not. The causal pies can be found in column one (see label). Columns two and three indicate if the individual has been exposed to each measured causal component (*A* and *O*, where *A* = 1 indicates individuals represented in the corresponding SCC models have been exposed). Columns four and five for population 1 and columns eight and nine for population 2 show the number of individuals in the example dataset who developed active-TB (*T* = 1) and who did not (*T* = 0), respectively. The sum of columns four and five for population 1 and eight and nine for population 2 is the total number of individuals exposed to each causal pie for each population. Finally, column seven for population 1 and eleven for population 2 is the risk ratio (RR) for each pie calculated using *S_1_* as the reference group

**Table 3:** The initial level of certainty, according to GRADE, differs between randomised controlled trials (RCTs) and nonrandomised studies (NRSs)

Type of evidence corresponding to initial level of certainty	Level of certainty	Definition of level of certainty			
Randomised controlled trials (RCTs)	High (four plus: ⊕ ⊕ ⊕ ⊕)	We are very confident that the true effect lies close to that of the estimate of the effect			
	Moderate (three plus: ⊕ ⊕ ⊕ ○)	We are moderately confident in the effect estimate: The true effect is likely to be close to the estimate of the effect, but there is a possibility that it is substantially different			
Nonrandomised studies (NRSs)	Low (two plus: ⊕ ⊕ ○○)	Our confidence in the effect estimate is limited: The true effect may be substantially different from the estimate of the effect			
	Very low (one plus: ⊕ ○○○)	We have very little confidence in the effect estimate: The true effect is likely to be substantially different from the estimate of effect			
Domains that may downgrade or upgrade (for observational evidence) a level of certainty					
Downgrade	Large effect + 1 Large + 2 Very large	Dose response + 1 Evidence of a gradient	All plausible residual confounding would: + 1 reduce a demonstrated effect + 1 suggest a spurious effect if no effect was observed		
Upgrade	Risk of Bias −1 Serious −2 Very serious	Inconsistency −1 Serious −2 Very serious	Indirectness −1 Serious −2 Very serious	Imprecision −1 Serious −2 Very serious	Publication bias −1 Likely −2 Very likely

The level of certainty indicates the confidence of investigators that the estimated effect is close to the true causal effect. GRADE provides domains that may upgrade or downgrade the level of certainty. Based on tables in [38]

Concerns about directness, inconsistency, imprecision and publication bias may reduce certainty. Directness refers to how closely the research evidence relates to the research question of interest, with different study populations (such as available evidence only focusing on adults, rather than children) or the use of surrogate outcomes being examples of ‘indirectness’. Inconsistency reflects differences in the effect size across studies (often identified through high levels of heterogeneity in a meta-analysis) which cannot be adequately explained. Imprecision occurs when effect estimates have wide confidence interval. Publication bias may arise if studies with a positive or exciting result are more likely to be published than those without a large association

**Table 4:** Summary of utilisation of each Bradford Hill (BH) viewpoint by each causal assessment approach: BH viewpoints, directed acyclic graphs (DAGs), sufficient-component cause models and GRADE methodology. Based on comparative analysis of causal assessment approaches

	Strength of association	Consistency	Specificity	Temporality	Dose-response	Plausibility	Coherence	Experiment	Analogy
Bradford Hill viewpoints	A strong association between an exposure and outcome indicates that the association is less likely due to something other than causality	Consistent observations of associations in different settings or populations indicate that the associations are less likely due to something other than causality	Evidence of specificity (one-to-one relationship) indicates that the association is less likely due to alternative variables (confounding) but absence of specificity does not undermine causality	Temporality is necessary for a causal argument to be made but may not always be clear, particularly with exposures that have an incubation period	Similar to strength of association, evidence of a dose-response relationship indicates that the association is less likely due to confounding	Critically evaluating plausible explanations for an association, other than causality, may strengthen a causal argument	Coherence is determined by how well assumptions about the causal relationship fit into existing theory	An association observed in an experiment provides strongest evidence that the association is not due to something other than causality	Associations between analogous exposures and outcomes indicate a similar causal mechanism and may strengthen a causal argument
Directed acyclic graphs	DAGs facilitate bias analysis which encourages articulating plausible confounding variables. Though DAGs cannot represent the size of an association, they can be used to consider the degree and implications of unmeasured and residual confounding	DAGs and SCC models provide a framework to elucidate the transportability of effect estimates. Transportability may be impacted by confounding structures in different settings or if the characteristics of different settings interact with the exposure. This may be useful for developing a causal explanation, which may then increase confidence in causality	DAGs cannot be used to articulate specificity, but they can be used to identify falsification outcomes (i.e. an outcome which cannot be plausibly associated with the exposure unless confounded) or falsification exposures (the opposite). The absence of a relationship between an exposures/outcomes and falsification variables are used to examine residual or unmeasured confounding and thus increase confidence in causality	DAGS can be used to articulate the potential for reverse causality which may undermine temporality	DAGs can be used to articulate confounding variables relevant to the relationship understudy. A high number of the confounding variables may undermine the relevance of a dose-response relationship in causal inference	SCC models and DAGs make the assumptions behind a causal relationship explicit, making it easier to consider the plausibility of the evidence and relationship	DAGs and SCC models are not helpful for considering coherence	DAGs can be used to articulate the extent to which exposure in certain study designs, such as natural experiment, resembles random exposure	DAGs and SCC models do not account for analogous relationships in their assessment, but analogous relationships may be part of developing the assumptions and theories encoded in the diagrams
Sufficient-component cause models	SCC models help to visually understand the impact prevalence of the outcome in the reference group has on the observed association		Specificity arises when a causal component is both necessary and sufficient to produce the outcome. SSC’s multifactorial nature illustrate the rarity of specificity	Time may be a component of a sufficient cause. Indicates a latent period that contributes to the outcome being observed	The unknown and unmeasured variables in SCC models limit their utility in understanding a dose-response relationship			Because unknown variables may differ between SCC models, they have limited utility for considering exchangeability between comparison groups	
GRADE methodology	GRADE provides guidance for what may be considered a large association. Upgrades NRSs if a large effect size is observed across a body of evidence	GRADE methodology underscores that consistent effect estimates, as described by Bradford Hill, may not give more confidence in causality as it could be due to the same bias. Rather, unexplained inconsistency (heterogeneous effect sizes) reduces confidence about the effect of the exposure on the outcome	GRADE does not take specificity into account, although it may be incorrectly conflated with indirectness	Evidence that proves participants were exposed before the outcome was recorded (such as an RCT) is graded higher than evidence that does not	GRADE suggest upgrading NRSs if a dose-response gradient is present because, alongside a strong effect, it indicates that the effect is less likely due to residual confounding	GRADE upgrades for adjustment for plausible confounding, but not plausibility of relationship	Coherence may be incorrectly conflated with indirectness, but GRADE does not account for coherence	Evidence from experimental studies graded higher than from non-experimental studies	Evidence of effect of exposure on analogous outcomes may prevent downgrading evidence, but this is more to do with applicability of surrogate outcomes rather than analogy as Bradford Hill described it

**Table 5:** Summary of conclusions. Interpretation of each BH based on mapping of DAGs, SCC models and GRADE

Bradford Hill viewpoint	Summary of comparisons	Implications for causal assessment
Strength of association	Bradford Hill argued that the stronger an association the less likely it could be explained by confounding, but did not make clear what should be constituted as strong. DAGs and SCC models can be used to consider how other variables might impact investigators’ confidence in a strong association and the extent to which it should be relevant to causal assessment. This includes the impact of several confounding variables or unknown and unmeasured confounding variables depicted by DAGs and the impact of competing causes depicted by SCC models, respectively. GRADE suggests potential thresholds for what constitutes a strong association	Strength of association should be considered in relation to potential residual confounding from unknown or unmeasured variables
Consistency	DAGs highlight that transportability (using the causal effect in one context to make causal inferences about a different population) issues may emerge due to differences in the confounding structures. SCCs illustrate that differences in prevalence of competing causes may result in variable effect sizes. GRADE draws attention to the importance of focusing on unexplained statistical heterogeneity (unexplained effect sizes that differ between populations)	A distinction needs to be made between different types of consistency namely transportability and unexplained statistical heterogeneity. Factors that may undermine transportability to another population may not undermine the causal relationship in that population. However, unexplained statistical heterogeneity may be used as evidence against a causal relationship
Specificity	One potential reason for specificity helping in causal assessment is that confounding cannot account for a specific relationship. DAGs can be used to extend this thinking to identify falsification exposures and outcomes. GRADE and SCC models reinforce Bradford Hill’s understanding of specificity, which is that a lack of specificity does not help with causal assessment	Specificity itself is rare and generally unhelpful in epidemiology. Falsification exposures or outcomes may strengthen evidence for a causal relationship, but may be difficult to identify
Temporality	DAGs explicitly incorporate the temporal ordering of variables and can be used to identify the potential biases due to reverse causality. Causal pies do not provide more insight, while GRADE privileges RCTs where the exposure necessarily precedes the outcome	Unchanged
Dose-response	Bradford Hill did not provide detailed explanations for how dose-response strengthened the evidence for causality. Similar to their use in strength of association, DAGs can be used to identify confounding variables which may create a spurious dose-response relationship. SCCs do not explicitly consider dose-response. GRADE currently uses the presence of a dose-response gradient to upgrade the certainty for a causal relationship	Dose-response is considered in both BH viewpoints and GRADE. However, it may not add as much to causal assessment as is commonly assumed, particularly if the impact of confounding variables is not considered alongside a dose-response gradient
Plausibility	DAGs and causal pies make assumptions about causal relationships explicit, thus they should be built upon plausibility. This transparency allows the plausibility of those assumptions to be interrogated by others. This, as well as the certainty assessed using GRADE, may provide evidence for the plausibility of the assumptions made in causal assessment	Plausibility can be formally encoded within DAGs to articulate the causal chain and in SCC models to articulate causal mechanisms, such as interaction between variables
Coherence	DAGs and causal pies do not typically consider coherence. GRADE does not consider coherence either, though it has been confused with indirectness. In practice, it is poorly delineated from plausibility	Utility not clearly supported
Experiment	Bradford Hill argued that experiment was the most important viewpoint for assessing causality. DAGs may help identify exchangeable groups (e.g. instrumental variables). SCC models do not explicitly consider experiments. GRADE privileges RCTs but does not discriminate between natural experiment studies and other NRSs	Consistent with what Bradford Hill argued, genuine experiments (trials), as well as quasi-experiments, can substantially strengthen causal inference
Analogy	Certainty in causality of analogous relationships or in causality of analogous outcomes may strengthen a causal argument or may be useful in developing assumptions about relationship, however these are not embedded into DAGs or SCC models. GRADE considers analogous exposures within the body of evidence, but not whether assumptions about analogous relationships can be transported to the causal relationship under study	Utility not clearly supported
